# Effect of *S. cerevisiae* strain KA500 supplementation on feed performance, feed efficiency, and digestion ability in feedlot buffaloes

**DOI:** 10.3389/fvets.2024.1397608

**Published:** 2024-07-16

**Authors:** Maiara dos Santos Ferreira, Welligton Conceição da Silva, Ancelmo Rodrigues Cunha, Ercvania Rodrigues Costa, Ícaro dos Santos Cabral, Salatiel Ribeiro Dias, Ronaldo Francisco de Lima

**Affiliations:** ^1^Institute of Social Sciences, Education and Zootechnics, Federal University of Amazonas, Parintins, Amazonas, Brazil; ^2^Postgraduate Program in Animal Science (PPGCAN), Institute of Veterinary Medicine, Federal University of Para (UFPA), Federal Rural University of the Amazônia (UFRA), Brazilian Agricultural Research Corporation (EMBRAPA), Castanhal, Brazil; ^3^Institute of Biodiversity and Forests, Federal University of Western Pará, Santarém, Pará, Brazil; ^4^Postgraduate Program in Graduate Program in Society, Nature and Development (PPGSND-UFOPA), Santarém, Brazil

**Keywords:** buffalo calves, performance, probiotic, *Saccharomyces cerevisiae*, buffalo

## Abstract

Live yeasts have favorable characteristics for use in animal feed, and may become a beneficial tool to improve digestive efficiency in buffaloes (*Bubalus bubalis*). The productive performance, feed efficiency, and digestion ability of buffaloes fed diets supplemented with yeast (*Saccharomyces cerevisiae* strain KA500) were evaluated. Eighteen male Murrah buffaloes, with initial weight 250 ± 31 kg (mean ± standard deviation), and aged approximately 12 months, were randomly assigned to one of two treatments. The treatments included experimental feed containing 10 g of the live yeast capable of forming 2 × 10^10^ colony forming units (CFU) and control (feed with no added yeast). The daily weight gain tended to be lower (*p* = 0.07) in buffaloes supplemented with yeast. There was a reduction in daily dry matter intake (DMI) and in % yield of live weight in buffaloes supplemented with yeast. There was no effect of live yeast supplementation on weight gain/kg dry matter intake, height at withers or rump, body condition score, total weight gain, carcass yield, plasma urea nitrogen concentrations, purine derivatives, and plasma glucose concentrations. The digestibility of dry matter (DM) and organic matter (OM) were lower (*p* < 0.05) with the supplementation of live yeast, although live yeast supplementation did not affect the digestibility of neutral detergent fiber (NDF) and non-NDF OM. The strain and dosage of live yeast used did not have a positive effect on buffalo performance and digestibility of dietary nutrients.

## 1 Introduction

Yeasts are unicellular eukaryotic organisms of the kingdom Fungi that ferment carbohydrates (1–3). Yeasts have been used in animal feed for more than 100 years, and the use of yeasts in animal feed has increased in recent years. There are more than 500 cataloged species of yeasts, and among them the most studied in animal nutrition is *Saccharomyces cerevisiae*, which has approximately 4000 registered strains (4). The observed responses regarding the use of *S. cerevisiae* in animal feed depend on the dosage offered to the animal, the type of strain used, the animal’s diet, feeding management, and the animal’s physiological characteristics (1, 5, 6).

Commercially available products containing yeasts used in ruminant nutrition are composed of a mixture of live cells or mixtures of different proportions of living and dead yeasts in the presence of the culture medium, called yeast culture. These products are based on *S. cerevisiae*, and differ in the chosen strains and other characteristics, such as concentration (colony forming units, CFU/g), cell viability, and processing. As different products differ in these characteristics, discrepant effects of such supplements are found in different studies.

Although there are many possible mechanisms of action for yeast, the action of yeast with regards to its effects on performance of ruminants is still not sufficiently clear. Positive effects of live yeast supplementation on the ruminal environment have been attributed to changes in the microbial population, providing an increase in cellulolytic bacteria, and bacteria that consume lactic acid (7, 8), and prevent the reduction of ruminal pH (9).

Yeasts are unicellular microorganisms that act in different biological processes, such as fermentation. In general, yeasts are involved in the production of volatile fatty acids, which are considered the main source of energy for buffaloes. In addition, yeasts help to prevent acidification in the rumen, as well as producing microbial proteins that are subsequently absorbed by the animal, contributing to the animal’s proper nutrition (6).

As for the specificity of the different buffalo diets, it is essential to take into account the fact that buffalo are herbivores and have a digestive system adapted to fermenting fibers. In general, their diet is based on grasses and leaves found in different places, especially in flooded areas. Supplementing cattle with specific strains of live yeasts has resulted in increased digestibility of (mainly low quality) fiber (10, 11) and increases in dry matter consumption (12, 13). Furthermore, Chaucheyras-Durand et al. (7), reported that the main beneficial effect of live yeasts with regards to fiber digestibility is the ability of living yeast cells to reduce ruminal oxygen, which improves the ruminal environment and allows other microorganisms to colonize the rumen and digest fiber.

Although there are several studies using yeast supplementation in the bovine diet, research on the use of yeast in the buffalo diet is scarce. However, some studies on yeast supplementation in the diet of buffalo calves have found signs of increases in the bacterial ruminal population through modified production of short chain fatty acids, lower acetate and propionate ratios (14), and improved feed efficiency in young animals consuming diets based on grass hay (15, 16). Thus, the objective of this study was to evaluate the effect of *S. cerevisiae* strain KA500 supplementation on feed performance, feed efficiency, and digestion ability in feedlot buffaloes.

## 2 Materials and methods

### 2.1 Ethical aspect

This study was submitted to and approved by the Ethics Committee for the Use of Animals (CEUA) of the Universidade Federal do Amazonas (UFMA), under number 014/2017.

### 2.2 Animals

Eighteen male, uncastrated Murrah buffaloes with initial body weights of 250 ± 31 kg (mean ± standard deviation) and 12 months of age were individually fed at 6:00 a.m. and 5:00 p.m., under a tie stall total confinement regime. Refusals were weighed, sampled and the rest discarded. Tie stalls had sawdust beds and individual troughs, and buffaloes had unrestricted access to water. The experimental period lasted 52 days, including 14 days of acclimatization, and an experimental period of 38 days.

### 2.3 Experimental design

There were nine blocks of two animals each, according to weight. In each block, one animal received the treatment and the other did not. Forming two groups of 9 animals each, one treated and one not. The groups were divided into yeast 250.1 ± 31.8 and control 245.8 ± 29.7. The experiment consisted of a randomized block design, adjusted to covariate, and included repeated measures over time. The two treatments included feed with 10 g of yeast, or feed without added yeast (control). The yeast was composed of the strain *S. cerevisiae* KA 500 capable of providing consumption of 2.0 × 10^10^ CFU of microorganisms per buffalo.

The buffaloes in both groups received the same diet during the 14 day acclimatization period. The acclimatization diet consisted of (based on the dry matter) 60% elephant grass silage of the Cameron variety (*Pennisetum purpureum* Schum), 12% soybean meal, 13% ground corn, 13% cupuaçu meal, and 2% mineral and vitamin complex. During the experimental period, the buffaloes were fed a diet consisting of (based on the dry matter) 29.2% elephant grass silage Cameron variety, and 70.8% concentrate (Table 1). The grass was harvested manually and minced in a stationary forage chopper, adjusted for a cut size of 4.5 mm. The silage was sealed for 30 days prior to delivery to the buffaloes. The mixing of the feed ingredients was performed immediately before buffaloes were fed, and each meal was sufficient to result in leftovers of approximately 10%.

**Table 1:** Composition of the diets fed to the buffaloes, including ingredients and nutrients.

Description	DM (%)
Elephant grass silage	29.2
Ground corn	23.1
Cupuaçu meal	21.8
Soybean hull	16.3
Soybean meal	7.2
Minerals and vitamins^1^	1.6
Urea	0.8
Crude protein (CP)	15.6
NDF^2^	36.8
Ethereal extract (EE)	6.4
Ash	3.4
NFC^3^	37.8
% of natural matter	
Dry matter	50.1

^1^Minerals and vitamins: 18.5% Ca; 15.0% P; 3.0% Mg; 3.0% S; 240 ppm Co; 3,000 ppm Cu; 80,000 ppm Mn; 12,000 ppm Zn; 90 ppm Se; 180 ppm I; 8,000,000 UI/kg Vit. A.; 2,000,000 UI/kg Vit. D; 50,000 UI/kg Vit. E. ^2^NDF = neutral detergent fiber; ^3^NFC = Non-fibrous carbohydrates = 100 – (CP + NDF + EE + Ash).

### 2.4 Bromatological analysis

The daily intake of dry matter (DM) and nutrients was calculated by measuring what was offered and subtracting the daily leftovers during days 10 to 14 of the acclimatization period, and daily throughout the experimental period. Samples of the ingredients and leftovers from each buffalo were collected daily and frozen. Equivalent quantities of natural matter from the daily samples were combined per week and were pre-dried in a ventilated oven for 72 h at 55°C, sieved with a 1 mm sieve in a Thomas-Willey type mill, and a sub-sample was dehydrated at 105°C for 24 h for determination of the DM content.

Crude protein (CP) was analyzed using a Micro Kjeldahl type steam distiller (17), and the ethereal extract (EE) was analyzed according to AOAC methods (18). Ashes were analyzed by incineration of the sample at 550°C for 8 h. The NDF concentration was analyzed using a TE-149 fiber determiner (Tecnal Equipamentos para Laboratórios, Piracicaba, SP), using amylase. Body weight, body condition score, chest circumference, and height at the withers and rump of buffaloes were determined on days 13 and 14 of the acclimatization period, and every 7 days of the experimental period. The heights were determined with the help of a graduated Lydtin cane at the dorsal end of the spine at the third thoracic vertebra (at the withers) and at the highest point of the sacral bone. The thoracic perimeter was determined caudally at the thoracic limbs. The body condition score (BCS) was visually assessed on a scale of 1 to 5, with a score of 1 being representative of lean, and a score of 5 being representative of fat, by three independent evaluators to obtain the mean per buffalo. The daily weight gain was calculated weekly in the experimental period as the difference between the pairs of interval determinations of 7 days.

Buffalo performance was evaluated up to the 35th day of the experimental period, and digestion parameters were evaluated, and blood samples were collected, on days 36 to 38 of the experimental period.

The apparent digestibility of total dry matter (DM), organic matter (OM), neutral detergent fiber (NDF), and non-NDF organic matter was determined on the 36th day of the experimental period by measuring fecal production by total fecal collection, carried out for 24 h.

After collection, fecal samples were dehydrated and the NDF and ash concentration were determined as described above. The percentage digestibility of these components was calculated as the amount consumed minus the amount excreted in the feces, divided by the amount consumed multiplied by 100.

### 2.5 Urine evaluation

Total urine collection was performed for 24 h at the same time as total feces collection. Urine was collected in buckets and stored in 20 liter plastic containers containing 200 mL of 20% sulfuric acid. After 24 h, the amount of urine was measured, and a sub-sample of approximately 50 mL was collected, and then diluted in 4% sulfuric acid solution (4, 1, acid, urine), and frozen at −20°C for measurements of concentrations of allantoin and creatinine. The procedure described by Chen and Gomes (19) was used for the allantoin analysis. A laboratory kit was used for the creatinine analysis (Creatinina. Doles Reagentes para Laboratório Ltda., Goiânia, GO).

### 2.6 Blood collection

Samples of jugular blood were collected on day 30 of the experimental period. Blood samples were first obtained immediately before feeding (considered time zero), and blood samples were subsequently collected every 3 h for a period of 24 h. Blood was sampled in evacuated tubes containing EDTA for analysis of plasma urea nitrogen (PUN) and in tubes containing potassium fluoride for analysis of plasma glucose. Plasma was obtained by centrifugation at 2,118 × *g* for 10 min and stored at −20°C. The PUN and glucose concentrations were measured using the colorimetric-enzymatic method (Ureia 500 e Glicose Enzimático Doles Reagente para Laboratórios Ltda., Goiânia, GO).

### 2.7 Masticatory activity

The masticatory activity was measured on day 28 of the experimental period by visual observation of the oral activity of each buffalo every 5 min per 24 h. The oral activities considered were food intake, rumination, and leisure. The chewing time in minutes per day was defined as the sum of the time spent on ingestion and rumination. Also, on day 28 of the experimental period, the time of first ingestion was evaluated.

### 2.8 Slaughtering the animals

On the 38th day of the experimental period, the buffaloes were fasted for 12 h, weighed, and then slaughtered at the municipal slaughterhouse. Measurements of carcass yield (as a percentage) of each buffalo was calculated as the relationship between carcass weight and live weight of animals and multiplied by 100.

### 2.9 Statistical analysis

The variables measured over time were analyzed as repeated measures using the SAS MIXED procedure (20). The covariance structure used was defined by the Akaike information criterion, among auto regressive order 1, composite, and unstructured symmetry. The statistical model was:

$\text{Yijk}=\mu +CV+B_{i}+L_{j}+T_{k}+{LT}_{jk}+\text{eijk}$

in which: μ = average overall; CV = covariate (measurement of the same variable at the end of the acclimatization period); Bi = block effect (i = 1 to 9); Lj = treatment effect (j = with or without yeast); T_k_ = time effect (k = day for variables evaluated over days, or hour for the blood variables evaluated during the daytime hours); LT_jk_ = interaction between treatment and time; e_ijk_ = residual error.

The mean square for the nested buffalo effect under treatment was used as an error measure to evaluate the treatment effect. The weight gain per week and the PUN concentration were analyzed using the same model, but without the covariant term. The variables measured once during the experimental period were analyzed using the same model without the effects of the covariate, time, and their interaction with treatment. Probability values below 0.05 were considered significant, probability values below 0.10 were considered a trend, and probability values below 0.15 were considered a weak trend.

## 3 Results

The data on dry matter intake (DMI), feed efficiency in kilograms per kg of dry matter intake, plasma urea nitrogen (PUN) concentrations, plasma glucose concentrations, the allantoin creatinine concentration, allantoin creatinine ratio of urine, and morphometry of buffaloes supplemented with live yeast (*S. cerevisiae*) or not (control) are presented in Table 2. Although not statistically different, yeast supplementation reduced the daily dry matter intake and percentage of live weight of buffaloes.

**Table 2:** Dry matter intake (DMI), food efficiency, plasma urea nitrogen (PUN) concentration, plasma glucose concentration, concentration of purine derivatives, allantoin:creatinine ratio, and morphometry of buffaloes supplemented with live yeast (*S. cerevisiae*) or not (control).

Description	Yeast	Control	SE	Time	Yeast	Time*Yeast
DMI, kg/d^1^	6.8	7.0	0.12	<0.01	0.18	0.20
DMI, % BW^2^	2.59	2.65	0.080	<0.01	0.61	0.41
Efficiency, kg gain/kg DMI	0.115	0.128	0.0126	<0.01	0.49	0.19
PUN, mg/dl	17.1	17.0	0.82	<0.01	0.94	0.37
Glucose, mg/dl	87.4	90.5	2.37	<0.01	0.38	0.97
Allantoin, g/d	2.5	2.8	0.43		0.63	
Creatinine, g/d	2.7	2.9	0.57		0.81	
Allantoin: Creatinine	1.21	1.16	0.215		0.86	
Thorax, cm	287	283	3.8	<0.01	0.47	0.20
Rump, cm	121	122	0.3	<0.01	0.02	0.28
Withers, cm	116	115	0.3	<0.01	0.25	0.27
^3^BCS, 1 a 5	3.8	3.7	0.12	<0.01	0.52	0.96

^1^DMI = Dry matter intake in kg per day. ^2^DMI, %BW = Dry matter intake in percentage of body weight.

The efficiency in gain/kg CMS, and the morphological measurements of height at the withers and rump, and thoracic perimeter, as well as body condition score (BCS) were not affected by yeast supplementation (Table 2). The mean BCS values were 3.8 and 3.7 for buffaloes supplemented with yeast and buffaloes in the control treatment, respectively.

**Figure 1:**
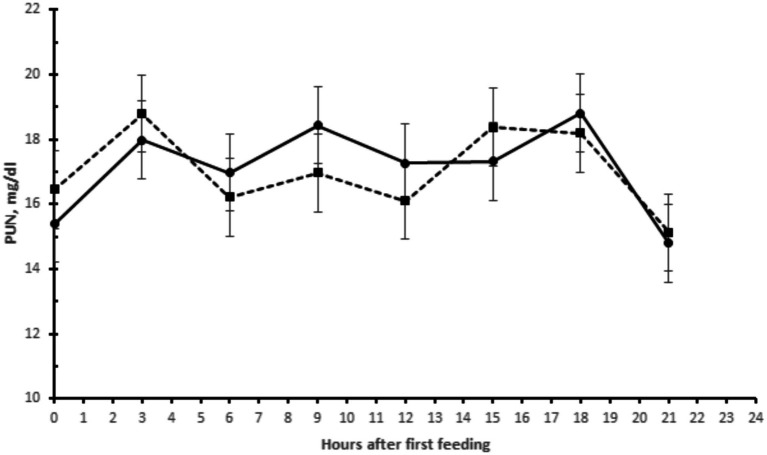
Plasma urea nitrogen (PUN) in buffaloes supplemented with live yeasts (- • -) or not (control) (-▪-).

No effect of yeast supplementation on plasma urea nitrogen (NUP) concentrations was found (Table 1 and Figure 1). The mean NUP concentration, assessed throughout the day, was between 14.5 and 19.0 mg/dL. The highest NUP concentrations were found 3 h after the morning and afternoon meals.

Purine derivatives (allantoin and creatinine) were not affected by yeast supplementation (Table 2). The microbial protein synthesis estimated by the relationship between allantoin and creatinine concentration in urine was higher when buffaloes were supplemented with yeast, although this was not statistically significant.

**Figure 2:**
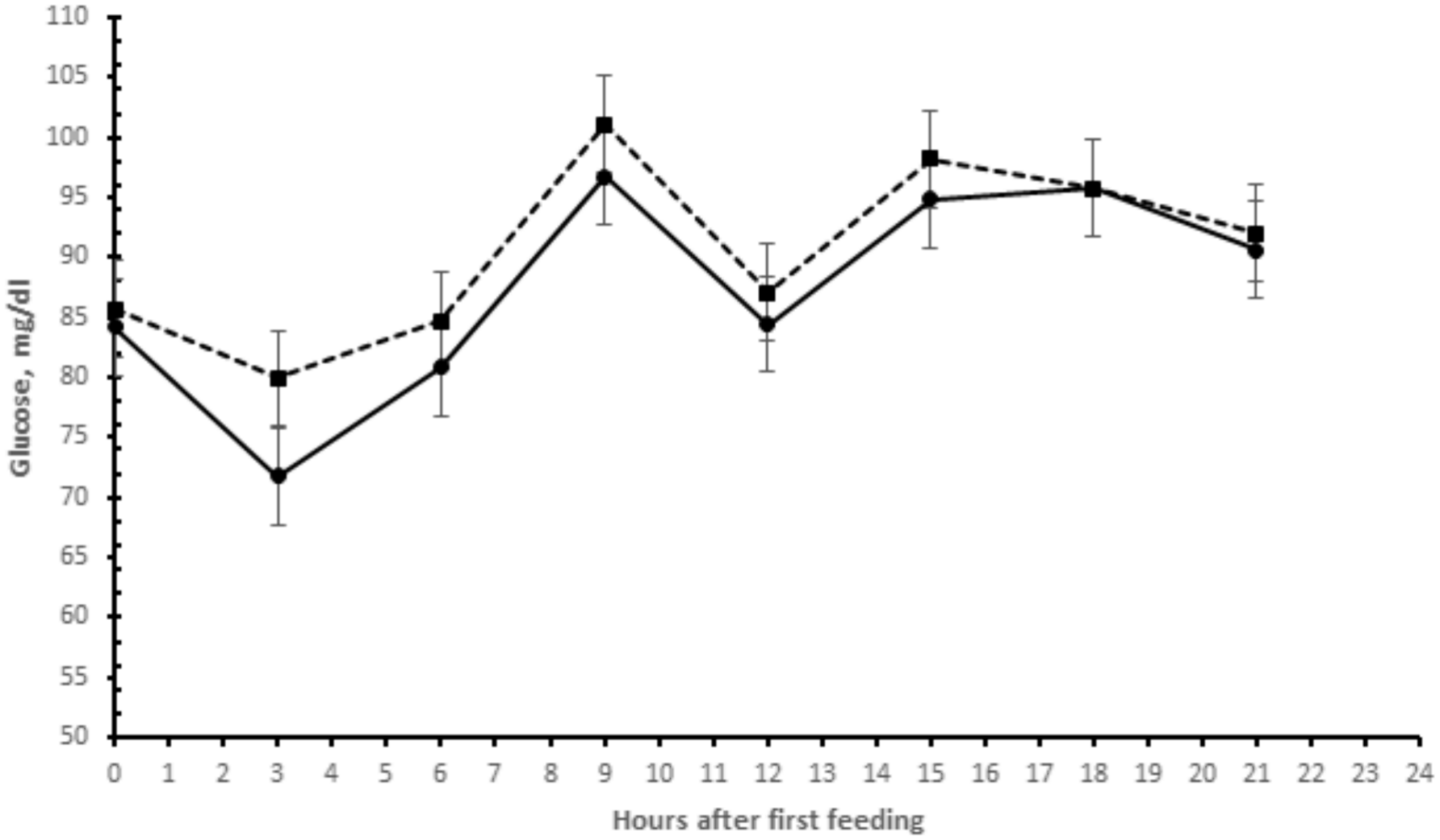
Plasma glucose concentrations of buffaloes supplemented with live yeasts (- • -) or not (control) (-▪-).

There were no significant differences in plasma glucose concentrations in buffaloes supplemented with yeast or buffaloes in the control group (Table 2). Plasma glucose concentrations were higher 8 h after the first feeding (Figure 2).

Dry matter digestibility (DMd) and organic matter digestibility (OMd) were lower (*p* < 0.05) in buffaloes supplemented with yeast (Table 3). However, the digestibility of neutral detergent fiber (NDFd) and digestibility of non-NDF organic matter (nNDFOMd) were not affected by yeast supplementation. Thus, in this study, supplementing buffaloes with live yeast did not result in beneficial changes in the digestion of fiber.

**Table 3:** Apparent digestibility of nutrients in the total digestive tract of buffaloes supplemented with live yeasts or not (control).

Description	Yeast	Control	SE^1^	*p*-value
DM^2^	54.3	58.3	1.29	0.05
OM	53.6	58.0	1.38	0.05
NDF	18.6	22.5	1.06	0.52
nNDFOM	35.08	35.5	3.30	0.93
Ethereal extract	84.9	81.9	4.14	0.62

^1^SE = Standard error. ^2^DM = Dry matter. OM = organic matter. NDF = Neutral detergent fiber. nNDFOM = Non-NDF organic matter.

Data on the performance of buffaloes supplemented with live yeasts or not (control) is presented in Table 4. There was no effect of yeast supplementation on total buffalo weight gain. However, there was a tendency (*p* = 0.07) for daily weight gain to be lower in buffaloes supplemented with yeast than in buffaloes in the control treatment.

**Table 4:** Performance of buffaloes supplemented with live yeasts or not (control).

Description	Yeast	Control	SE^1^	*p*-value
IBW^2^	250.1	245.8	3.51	0.42
FBW^3^	282.3	284.7	3.86	0.68
Carcass weight, kg	125.5	124.3	3.64	0.76
Carcass yield, %	44.5	43.5	0.776	0.29
Total gain, kg	32.1	38.9	3.28	0.19
DWG^4^, kg/d	0.879	1.089	0.1132	0.07

^1^SE = Standard error. ^2^IBW = Initial body weight. ^3^FBW = Final body weight. ^4^DWG = Daily weight gain.

The carcass yields found in this experiment were 44.5 and 43.5% for buffaloes supplemented with yeast and buffaloes in the control treatment, respectively (Table 4). This indicates that carcass yields increased when buffaloes were supplemented with yeast in relation to the control treatment, although this difference was not statistically significant.

There was no effect of yeast supplementation on time spent chewing (Table 5). The time taken for first intake in minutes was not affected by yeast supplementation.

**Table 5:** Oral activities of buffaloes supplemented buffaloes with live yeast or not (control).

Description	Yeast	Control	SE^1^	*p*-value
Rumination, min	576	571	17.8	0.84
Ingestion, min	180	187	14.3	0.74
Chewing, min^2^	756	757	22.1	0.96
Time First Ingestion, min	19	19	1.4	0.84

^1^SE = Standard error. ^2^Chewing = rumination + ingestion.

## 4 Discussion

Although we did not find a statistical difference in DMI, there was a reduction in consumption (Table 2), which agrees with Malik and Bandla (21) and Ullah et al. (22) that reported a reduction in dry matter intake (DMI) in buffalo calves supplemented with live yeast (*S. cerevisiae*). Already, Di Francia et al. (15) found no effect of yeast on dry matter intake in buffalo calves.

The mean BCS values were 3.8 and 3.7 for buffaloes supplemented with yeast and buffaloes in the control treatment, respectively, indicating that the buffaloes were in good body condition. The values of thoracic perimeter found in this study, both in buffaloes supplemented with yeast and buffaloes in the control treatment, were higher than the values reported in previous studies.

Evaluation of NUP concentrations is a way to monitor the efficiency of protein use in the diet, resulting in indicators of the nitrogen-energy ruminal balance. The fact that yeast supplementation did not affect the plasma NUP concentration can be explained by the lack of effect of yeast on the digestibility of dietary organic matter. A similar result was reported by El-Ashry et al. (23), who found that the urea concentration in plasma of lambs was not significantly affected by yeast supplementation. Buffaloes are less efficient at using N on a high protein diet. Naveed-ul-Haque et al. (24) observed an increase in PUN from 21.1 to 26.6 mg/dl when the dietary protein content of milk buffaloes increased from 11 to 14.2% based on dry matter.

This trend of lower weight gain can be explained by the reduction in the digestibility of organic matter. Growth-related responses to yeast supplementation varied from no significant effect on mean daily gain to increases of more than 20% (25). This could be due to the amount of yeast provided to buffaloes in this study, which was higher than the amount used in most previous studies, or this could be due to the strain used, the animal diet, feed management, the physiological characteristics of the buffaloes (1, 7), or thermal stress, which may have contributed to the reduction in CMS, affecting the daily weight gain. Differently from our study, that we found no difference between the treatment in plasma glucose levels, Singh et al. (26) reported increases in plasma glucose concentrations in buffaloes supplemented with yeast.

In this study, supplementing buffaloes with live yeast did not result in beneficial changes in the digestion of fiber. Several studies conducted in cattle have shown that strain type, diet, and yeast dosage may affect the effect of yeast supplementation on performance and nutrient digestibility in cattle. According to Calabro et al. (27), buffaloes are more capable of digesting feed with higher fiber contents. Thus, supplementing buffaloes with yeast may not have resulted in improvements in fiber digestibility, as buffaloes are already able to efficiently digest fiber.

Reddy (28) found no effects supplementing buffaloes with 5 g of yeast on nutrient digestibility of DM, OM, NDF. Kumar et al. (29) conducted an experiment with buffalo calves supplemented with *S. cerevisiae* strain CNCM I-1077 at a rate of 0.25 g/calf/day and found that yeast supplementation resulted in a significantly higher digestibility of DM, EE, OM, CP, ADF, and NDF, compared to the control diet. In another study by Kumar et al. (30) on the effect of yeast culture supplementation, buffaloes were supplemented with *S. cerevisiae* (0.5 g/buffalo/day; 1.5 × 10^8^ cfu/kg) and reported that dry matter intake (kg/100 kg body weight), CP digestibility, total digestible nutrients (TDN), nutrient digestibility, and crude fiber fractions were not affected by yeast supplementation as compared to the control diet.

Previous studies found that the addition of 0.25 to 5 g/buffalo/day resulted in improvements in the performance of buffalo calves (29). This suggests that the addition of 10 g/buffalo/day (2.0 × 10^10^ cfu/kg) of yeast (*S. cerevisiae*) in this study may have been high, which may limit consumption, reduce nutrient digestibility, and consequently affect the daily weight gain.

The final body weight (FBW), carcass yield (%), and carcass weight (kg) increased during the study period, but these increases were not statistically significant. The tendency for daily weight gain to be lower in buffaloes supplemented with yeast than in buffaloes in the control treatment can be explained by the reduction in the digestibility of organic matter. Growth-related responses to yeast supplementation varied from no significant effect on mean daily gain to increases of more than 20% (25). This could be due to the amount of yeast provided to buffaloes in this study, which was higher than the amount used in most previous studies.

The carcass yields increased when buffaloes were supplemented with yeast in relation to the control treatment, although this difference was not statistically significant. The carcass yields found in this study are smaller than those reported in most other studies, (49.9 to 51.7%) (31). The lower carcass yield found in buffaloes is a consequence, mainly, of the greater weights of leather and heads in these animals (32). In other cases, there is a large variation between animal weights, and the breed effect is confounded by the effect that body weight has on carcass yield. The lower carcass yield of buffaloes in our experiment can be explained by the lower age of the animals (approximately 14 months at slaughter).

The absence of effect, by yeast supplementation, on time spent with chewing and the time taken for first intake in minutes may be correlated with the digestibility of NDF, which also did not change with the supplementation of live yeasts. The higher or lower digestibility of NDF could cause an increase or reduction in the time of first intake, respectively. A greater digestibility of NDF would reduce the physical limitations of consumption (33), which would lead to a longer intake time. Devries and Chevaux (34) reported no effect of yeast on time spent on ingestion in dairy cows.

The limitations of this study were the length of time it took to evaluate the action of the yeasts and the number of animals, so we suggest that new studies be carried out with a larger number of animals, with more time to evaluate the yeasts and with more variables.

## 5 Conclusion

In conclusion, supplementing buffaloes with 10 g (2 × 10^10^ cfu/kg of viable cells) of *S. cerevisiae* strain KA500 did not significantly affect consumption, weight gain, and feed efficiency. There was a trend for daily weight gain to be lower in buffaloes supplemented with yeast, which can be explained by a reduction in organic matter digestibility. The dose of live yeast in buffalo diets appeared to be high, compared to the doses used in previous studies. However, yeast cultures do not act similarly when added to any diet. This study shows that the mode of action of yeasts is dependent on the strain and dosage used, diet, food handling, physiological characteristics of animals, and animal health. It is recommended that future studies investigate different dosages, with the addition of different yeasts or other potential additives that have potential effects on the digestion and metabolism of buffalo.

## Data availability statement

The raw data supporting the conclusions of this article will be made available by the authors, without undue reservation.

## Ethics statement

This study was submitted to and approved by the Ethics Committee for the Use of Animals (CEUA) of the Universidade Federal do Amazonas (UFMA), under number 014/2017. The study was conducted in accordance with the local legislation and institutional requirements.

## Author contributions

MF: Conceptualization, Data curation, Formal analysis, Investigation, Methodology, Software, Validation, Writing – original draft, Writing – review & editing. WS: Formal analysis, Investigation, Methodology, Validation, Writing – original draft, Writing – review & editing. AC: Data curation, Investigation, Methodology, Supervision, Validation, Writing – original draft, Writing – review & editing. EC: Conceptualization, Data curation, Investigation, Methodology, Supervision, Writing – original draft, Writing – review & editing. ÍC: Investigation, Methodology, Supervision, Writing – original draft, Writing – review & editing. SD: Data curation, Investigation, Methodology, Writing – original draft, Writing – review & editing. RL: Conceptualization, Data curation, Formal analysis, Funding acquisition, Investigation, Methodology, Project administration, Resources, Software, Supervision, Validation, Visualization, Writing – original draft, Writing – review & editing.
